# Prolyl-4-hydroxylase Α subunit 2 (P4HA2) expression is a predictor of poor outcome in breast ductal carcinoma in situ (DCIS)

**DOI:** 10.1038/s41416-018-0337-x

**Published:** 2018-11-09

**Authors:** Michael S. Toss, Islam M. Miligy, Kylie L. Gorringe, Abdulbaqi AlKawaz, Hazem Khout, Ian O. Ellis, Andrew R. Green, Emad A. Rakha

**Affiliations:** 1 Nottingham Breast Cancer Research Centre, Division of Cancer and Stem Cells, School of Medicine, The University of Nottingham, Nottingham City Hospital, Nottingham, UK; 2 Histopathology department, South Egypt Cancer Institute, Assiut University, Assiut, Egypt; 3 Histopathology department, Faculty of Medicine, Menoufia University, Menoufia, Egypt; 4 Cancer Genomics Program, Peter MacCallum Cancer Centre, Melbourne, Australia; 5 The Sir Peter MacCallum Department of Oncology, University of Melbourne, Parkville, Australia; 6 Department of Surgery, Breast Institute, Nottingham University Hospitals NHS Trust, Nottingham City Hospital, Nottingham, UK

**Keywords:** Breast cancer, Breast cancer, Breast cancer, Breast cancer

## Abstract

**Background:**

Extracellular matrix (ECM) plays a crucial role in tumour behaviour. Prolyl-4-hydroxlase-A2 (P4HA2) is a key enzyme in ECM remodelling. This study aims to evaluate the prognostic significance of P4HA2 in breast ductal carcinoma in situ (DCIS).

**Methods:**

P4HA2 expression was assessed immunohistochemically in malignant cells and surrounding stroma of a large DCIS cohort comprising 481 pure DCIS and 196 mixed DCIS and invasive carcinomas. Outcome analysis was evaluated using local recurrence free interval (LRFI).

**Results:**

High P4HA2 expression was detected in malignant cells of half of pure DCIS whereas its expression in stroma was seen in 25% of cases. Higher P4HA2 expression was observed in mixed DCIS cases compared to pure DCIS both in tumour cells and in stroma. High P4HA2 was associated with features of high risk DCIS including younger age, higher grade, comedo necrosis, triple negative and HER2-positive phenotypes. Interaction between P4HA2 and radiotherapy was also observed regarding the outcome. High P4HA2 expression was an independent prognostic factor in predicting shorter LRFI.

**Conclusion:**

P4HA2 plays a role in DCIS progression and can potentially be used to predict DCIS outcome. Incorporation of P4HA2 with other clinicopathological parameters could refine DCIS risk stratification that can potentially guide management decisions.

## Introduction

Currently, determination of behaviour and proper management of DCIS depends on conventional clinicopathological parameters including patient age, nuclear grade, mode of disease presentation either symptomatic or screen detected, tumour size and presence of comedo type necrosis.^1,2^ Despite the prognostic value of these factors, they remain insufficient to define risk of progression precisely. Moreover, these parameters classify more than 50% of DCIS in the high-risk group, however, the recurrence rate is currently around 15% with half of these being invasive.^3^ Therefore, a considerable percentage of DCIS patients are either over- or under-treated.^4^ Molecular characterisation based on hormonal receptors and HER2 status in addition to recently described multigene assays such as Oncotype DX DCIS score, have shown promising results to refine DCIS prognostic classification but the value of their application in routine practice remains unclear.^5–9^

The role of the DCIS microenvironment and the crosstalk between intraductal malignant epithelial cells and the various components of the extra-ductal structures in the progression of DCIS to invasive disease is undeniable.^10–14^ However, previous studies have indicated that the precise role of proteolytic enzymes in the extracellular matrix (ECM), such as matrix metalloproteinases (MMPs), in predicting risk of development of local recurrence is unreliable and trials for blocking these enzymes to limit tumour progression showed disappointing results.^15,16^

Collagen is the main constituent of ECM and usually forms a network around tumour cells.^17^ Collagen biosynthesis is a multistep process with several post-translational modifications. Prolyl-4-hydroxylases catalyse the formation of 4-hydroxyproline, which is essential for collagen triple helix formation and fibre stabilisation.^18^ Increased P4HA2 expression has been detected in many solid tumours, including oral cavity squamous cell carcinoma,^19^ papillary thyroid cancer,^20^ and invasive breast carcinoma (IBC).^21^ Interestingly, P4HA2 is differentially expressed between normal breast tissue and IBC.^22,23^ However, to the best of our knowledge, no previous study has addressed the role of P4HA2 in DCIS progression and its prognostic impact. In this study, we aimed to assess the expression of P4HA2 in a large cohort of DCIS to evaluate its clinicopathological and prognostic significance.

## Material and methods

### Study cohort

This study was carried out on a consecutive series of 776 primary pure DCIS cases diagnosed between 1990 to 2012 at Nottingham City Hospital, Nottingham, United Kingdom (UK). Supplementary Table 1 summarises the clinicopathological parameters of the study cohort. A series of 239 cases diagnosed as synchronous DCIS and IBC (DCIS-mixed) was also collected as a comparison set. The latter was selected with clinicopathological features comparable to the pure cohort to avoid any selection bias (Supplementary Table 1). Patients’ demographic data, histopathological parameters, management, including post-operative radiotherapy (RT) and development of local recurrence were collected. Local recurrence free interval (LRFI) was defined as the time (in months) between 6 months after the first DCIS surgery and occurrence of ipsilateral local recurrence (either as DCIS or IBC with or without ipsilateral nodal metastasis). Cases that underwent completion re-excision surgery within the first 6 months of the primary operation were not considered as recurrence. Patients developed contralateral disease following DCIS diagnosis were censored at the time of development of the contralateral cancer. Within a median follow-up period of 103 months (range 6–331), 83 cases (11%) developed a recurrence in the pure DCIS cohort compromising 30 DCIS (36%) and 53 IBC with or without DCIS (64%). Six recurrence events were developed after mastectomy and 11 events after management with breast conserving surgery (BCS) followed by adjuvant RT while the majority of the recurrences (*n* = 66) occurred after BCS alone.

### Immunohistochemistry

Tissue microarrays (TMAs) were prepared from DCIS cohorts. The TMA was constructed using a TMA GRAND MASTER 2.4-UG-EN MACHINE, using 1 mm punch sets. Cases with heterogeneous growth patterns or grades were sampled from all representative areas. For mixed cohort, a separate TMA from each component (DCIS and IBC) was constructed. In addition, whole tissue sections from 12 cases compromising 8 pure DCIS and 4 DCIS-mixed cases were assessed to evaluate heterogeneity and the pattern of P4HA2 expression in malignant breast tissue and adjacent normal tissue.

**Fig. 1:**
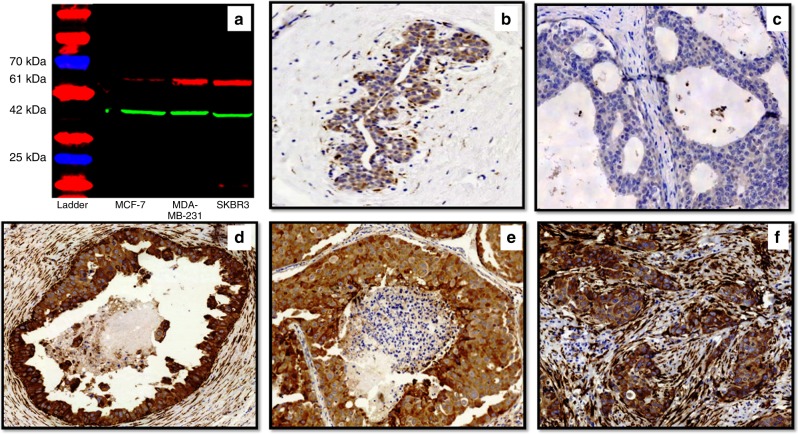
Anti-P4HA2 antibody validation and patterns of protein expression. **a** Western blot of mouse monoclonal anti-P4HA2 antibody showing a single specific band (upper red band) at expected molecular weight (61 kDa) in MCF-7, MDA-MB-231 and SKBR3 cell lysates. The lower green band represents the beta-actin (positive control) at 42 kDa molecular weight, **b** Normal breast duct (x20) shows faint cytoplasmic staining of P4HA2 in the normal epithelial cells. Occasional faint staining in the myoepithelial cells is also noticed. **c** Negative P4HA2 expression (x40) in a pure DCIS case; (**d**) strong expression of P4HA2 in tumour cells and surrounding fibroblasts (x20) in a pure DCIS case. **e**, **f** Expression of P4HA2 in a mixed case (x40) showing almost the same intensity within the tumour cells of DCIS component (**e**) and invasive component (**f**) while expression within the surrounding stromal fibroblasts is higher in invasive component (**f**)

Primary antibody specificity for mouse monoclonal P4HA2 antibody [CL0351, ab211527, Abcam, UK] was validated using western blot on whole cell lysates of MCF7, MDA-MB-231 and SKBR3 human breast cancer cell lines (obtained from the American Type Culture Collection; Rockville, MD, USA) as previously described.^21–23^ P4HA2 is expressed by epithelial tumour cells and specifically breast cancer cell lines as reported in Gilkes et al., Pan et al., and Xiong et al.,^21–23^ hence, these cell lines were used. P4HA2 antibody was used at a dilution of 1:500 which showed a single specific band at the predicted size of 61 KDa (Fig. 1a).

Expression of P4HA2 protein in DCIS was assessed by immunohistochemistry (IHC) using the Novocastra Novolink TM Polymer Detection Systems kit (Code: RE7280-K, Leica, Biosystems, UK). TMA and full-face sections (4 µm) were stained with mouse monoclonal P4HA2 (dilution 1:100), incubated for 16 h. Skin tissue was used as a positive control while a cerebral cortex tissue section was used as a negative control.

For molecular characterisation of the DCIS cohort, immunohistochemical staining of oestrogen receptor (ER) progesterone receptor (PR) and HER2 was carried out on the TMA sections (4 µm). For ER and PR, sections were stained on the Ventana Benchmark® ULTRA system (Tucson, Arizona, USA) using Ventana anti-ER (SP1) Rabbit Monoclonal Primary Antibody and anti-PR (1E2) Rabbit Monoclonal Primary Antibody as per the recommended protocol. The primary antibody was applied for 16 min at 37 °C followed by the OptiView HQ Linker for 8 min and the OptiView HRP Multimer for 8 min. Cases with nuclear staining of more than 1% of the tumour cells were considered positive. HER2 status was assessed using IHC staining (1:400, DAKO, no antigen retrieval) with HercepTest scoring method as previously published.^24,25^ Chromogenic in situ Hybridisation (CISH) was used to assess the *HER2* gene amplification to determine the final status of HER2 within equivocal cases. *HER2* gene amplification was deemed positive where there were six or more signals per nucleus or when clusters were identified in the tumour cell nuclei in more than 50% of tumour cells.^26^ Moreover, as hypoxia is reported to be the key driver for P4HA2 upregulation,^27^ the pure DCIS cohort was stained and scored for hypoxia inducible factor 1 alpha (HIF-1a) [EPR16897, ab179483, Abcam, UK] as previously described.^28^

### Scoring of P4HA2 expression

Cytoplasmic expression of P4HA2 in tumour epithelial cells and the surrounding stromal fibroblasts was assessed. Semi-quantitative Histo-score (H-score) was applied for cytoplasmic expression of P4HA2 in epithelial cells (staining intensity was multiplied by the percentage of representative cells in the tissue for each intensity, producing a range of values between 0 and 300).^29^ Cores containing < 15% tumour epithelial cells and/or stroma were excluded from the scoring. All scored cores showed representative areas of specialised stroma^30^ surrounding the malignant ducts. Cases with multiple cores (n = 180) were scored and the average was used as a final score. Cytoplasmic expression of P4HA2 within the stromal fibroblasts was assessed as percentage of positive cells. Determination of the expression intensity within the scanty cytoplasm of the slender shaped fibroblasts was challenging; thus, staining intensity was not scored. For mixed cohort, we scored each component, DCIS and IBC, separately. The cases were scored by 2 pathologists using multiheaded microscope. For dichotomisation of protein expression, cut-off points for either stromal or epithelial cells expression of P4HA2 were defined according to the conducted results from X-tile bioinformatics software (Yale University, version 3.6.1) based on LRFI in the pure DCIS cohort.^31^ High P4HA2 expression within tumour epithelial cells was considered when H-score was > 40 while expression in > 60% of the surrounding stromal fibroblasts was considered high expression.

### Analysis of P4HA2 mRNA expression in breast cancer

To emphasise the prognostic role of P4HA2 in breast cancer and given the lack of published data on the transcriptomic profiles of DCIS, the Molecular Taxonomy of Breast Cancer International Consortium (METABRIC) cohort,^32^ which comprises a large (n = 1980) well characterised cohort of IBC with comprehensive molecular characterization was used. *P4HA2* normalised gene expression (mRNA) was evaluated as a potential prognostic marker in the METABRIC dataset.

### Statistical analysis

Statistical analyses were performed using SPSS v21 (Chicago, IL, USA) for Windows. Student’s t test and analysis of variance (ANOVA) were used to correlate between *P4HA2* mRNA level as a continuous variable and other clinicopathological parameters in METABRIC data. Association with *P4HA2* mRNA expression and breast cancer specific survival (BCSS) was done using uni- and multi-variate analysis models after dichotomisation of expression into high and low based on the median value. Spearman’s Rho test was used to correlate between P4HA2 expression with the tumour epithelial and stromal cells. Association between P4HA2 expression and clinicopathological parameters as well as RT in pure DCIS was performed using Chi-square, Mann Whitney and Kruskal-Wallis tests. Wilcoxon signed rank test was used to compare the expression of P4HA2 between DCIS component and invasive component within the DCIS-mixed cases. Univariate survival analysis against LRFI was carried out using log rank test and Kaplan Meier curves. Multivariate analysis and the interaction between P4HA2 expression and RT was analysed using Cox regression model. For all tests, a two-tailed *p*-value of less than 0.05 was considered as statistically significant.

## Results

### Pattern of P4HA2 expression

The evaluation of full-face tissue sections demonstrated a rather homogenous distribution of P4HA2 expression either within the tumour epithelial cells or the surrounding specialised stroma especially in cases with homogenous pattern and grade, indicating representability of TMAs to assess P4HA2 expression in our cohort. Adjacent normal breast terminal ducto-lobular units showed negative or faint cytoplasmic staining of P4HA2. Occasional myoepithelial and inflammatory cells in a few cores were also stained. When present, P4HA2 was expressed in the cytoplasm of the epithelial tumour cells and surrounding fibroblasts.

After excluding of uninformative cores (they were randomly excluded due to loss of the cores during TMA construction or antigen retrieval, folded tissue during processing or cores containing < 15% tumour cells), the final number of cases suitable for scoring was 481 in pure DCIS and 196 in DCIS-mixed cohorts. P4HA2 expression showed a unimodal distribution. The median H-score for P4HA2 expression was 40 in pure DCIS (range 0–250), 50 in the DCIS component of mixed cases (range 0–280), and 40 in IBC component of the latter (range 0–280). For stromal expression, the median percentage of positive stromal cells was 30% in pure DCIS (range 0–95), 55% in the DCIS component of mixed cases (range 0–95) and 80% in the invasive component of the latter (range 0–95). Within the pure DCIS cohort, high P4HA2 expression was observed in 247/481 (51.4%) and 121/481 (25.2%) in tumour epithelial and surrounding stromal cells; respectively. There was a positive linear correlation between expression of P4HA2 within the epithelial cells and surrounding fibroblasts (*r* = 0.426, *p* < 0.0001, Spearman’s correlation).

The proportion of cases with high P4HA2 was greater in DCIS-mixed than pure DCIS, both within the tumour epithelial cells (54% of pure DCIS cases vs. 64% of DCIS mixed with IBC, *χ*^*2*^ = 8.6, *p* = 0.003) and stromal cells (25% for pure DCIS vs. 50% of DCIS mixed with invasion, χ2 = 39.3, *p* < 0.0001). Supporting this, similar observations results were observed when the data had been analysed in a continuous scale (*p* = 0.006 and *p* < 0.0001, for tumour epithelial cells and stromal cells, respectively). Although there was no statistically significant difference between P4HA2 expression within the tumour epithelial cells of DCIS component and invasive component of DCIS-mixed cases (*p* = 0.188), its expression within the stromal fibroblasts surrounding the invasive component was higher than those surrounding the DCIS component (*p* < 0.0001). Different patterns of P4HA2 expression within the pure DCIS and DCIS-mixed cohorts are shown in Fig. 1 (b-f). Supplementary Figure 1 shows some examples of P4HA2 expression around 40 H score.

### Significance of P4HA2 expression in pure DCIS

High expression of P4HA2 within the malignant epithelial cells and/or surrounding stromal fibroblasts in the pure DCIS was associated with various clinicopathological parameters characteristic of high risk DCIS (Table 1) including younger age at diagnosis, DCIS presented symptomatically, high nuclear grade, presence of comedo necrosis, ER negativity, PR negativity, HER2 positivity, and triple negative DCIS. Also, there was a positive association between high P4HA2 expression either within tumour epithelial cells or stromal cells and high HIF-1a expression. The majority of patients receiving post-operative adjuvant RT showed high P4HA2 expression as well. Analysis of continuous data of P4HA2 expression scores showed comparable results (Supplementary Table 2).

**Table 1:** Correlation between P4HA2 expression in DCIS malignant epithelial cells and stromal fibroblasts with different clinicopathological parameters in the pure DCIS cohort

Parameters	P4HA2 expression in tumour epithelial cells		*χ*^2^ (*p* value)	P4HA2 expression in stromal fibroblasts		*χ*^2^ (*p* value)
	Low (*N* = 234) *N*. (%)	High (*N* = 247) *N*. (%)		Low (*N* = 360) *N*. (%)	High (*n* = 121) *N*. (%)	
*Patient age*						
≤45 years	18 (8)	40 (16)	8.2	38 (11)	20 (17)	3.0
>45 years	216 (92)	207 (84)	**(0.004)**	322 (89)	101 (83)	(0.081)
*DCIS presentation*						
Screening	126 (54)	112 (45)	3.5	189 (53)	49 (40)	5.2
Symptomatic	108 (46)	135 (55)	(0.062)	171 (47)	72 (60)	**(0.020)**
*DCIS size*						
≤20 mm	103 (44)	101 (41)	0.4	158 (44)	46 (38)	1.2
>20 mm	130 (56)	144 (59)	(0.510)	200 (56)	74 (62)	(0.266)
*DCIS nuclear Grade*						
Low	51 (22)	10 (4)		52 (14)	9 (7)	
Moderate	73 (31)	52 (21)	49.4	98 (27)	27 (22)	6.4
High	110 (47)	185 (75)	**(** **<** **0.0001)**	210 (59)	85 (71)	**(0.04)**
*Comedo necrosis*						
Yes	130 (56)	191 (77)	25.6	226 (63)	95 (79)	10.1
No	104 (44)	56 (23)	**(** **<** **0.0001)**	134 (37)	26 (21)	**(0.001)**
*Estrogen receptor (ER)*						
Negative	24 (11)	93 (41)	50.9	75 (23)	42 (39)	10.9
Positive	190 (89)	132 (59)	**(** **<** **0.0001)**	256 (77)	66 (61)	**(0.001)**
*Progesterone receptor (PR)*						
Negative	65 (30)	121 (54)	25.0	127 (38)	59 (55)	8.7
Positive	149 (70)	103 (46)	**(** **<** **0.0001)**	203 (62)	49 (45)	**(0.003)**
*HER2 status* ^a^						
Negative	164 (81)	156 (69)	8.4	245 (77)	75 (69)	2.2
Positive	38 (19)	70 (31)	**(0.004)**	75 (23)	33 (31)	(0.141)
*Surgical management*						
Mastectomy	130 (56)	142 (58)	0.2	204 (57)	68 (56)	0.1
BCS	104 (44)	105 (42)	(0.669)	156 (43)	53 (44)	(0.928)
*Radiotherapy (RT)* ^b^						
Yes	29 (28)	42 (40)	3.5	43 (28)	28 (53)	11.3
No	75 (72)	63 (60)	**(0.04)**	113 (72)	25 (47)	(0.001)
*Molecular classes*						
Luminal/HER2–	147 (76)	98 (47)		198 (65)	47 (48)	
Luminal/HER2 +	24 (12)	28 (13)	45.9	37 (12)	15 (15)	10.2
ER-/HER2 +	11 (6)	36 (17)	**(** **<** **0.0001)**	33 (11)	14 (14)	**(0.017)**
Triple negative	11 (6)	48 (23)		37 (12)	22 (23)	
*HIF1-a expression*						
High	24 (15)	132 (66)	18.1	58 (21)	34 (36)	8.1
Low	141 (85)	68 (34)	**(** **<** **0.0001)**	213 (79)	60 (64)	**(0.004)**
*Ipsilateral local recurrence*						
Yes	17 (7)	39 (16)	8.5	47 (13)	9 (8)	2.8
No	217 (93)	208 (84)	**(0.004)**	313 (87)	112 (92)	(0.100)
*DCIS Type* ^c^						
Pure DCIS	234 (77)	247 (66)	8.6	360 (79)	121 (55)	39.3
DCIS with IBC	71 (23)	125 (34)	**(0.003)**	98 (21)	98 (45)	**(** **<** **0.0001)**

significant p values are in bold

*P4HA2* prolyl-4-hydroxlase alpha subunit 2, *DCIS* ductal carcinoma *in situ*, *HER2* human epidermal growth factor receptor 2, *BCS* breast conserving surgery, *IBC* invasive breast cancer, *HIF1-a* hypoxia inducible factor 1 alpha

^a^HER2 final status is achieved using combination of IHC and chromogenic in situ hybridisation (CISH)

^b^For patients treated with breast conserving surgery

^c^Including the cases in both cohorts; i.e. pure DCIS cohort (*n* = 481) + DCIS-mixed cohort (*n* = 196)

To validate the prognostic value of P4HA2 in IBC, the METABRIC cohort^32^ was used to assess the levels of *P4HA2* mRNA and correlate its expression with the clinicopathological variables and outcome. We considered mRNA expression data to be valid for comparison, as The Cancer Genome Atlas data (cBio, Provisional breast cancer data set, obtained February 2018) shows that *P4HA2* mRNA expression by RNAseq and protein expression by mass spectrometry in 70 IBC were significantly positively correlated (*p* < 0.0001, Spearman r = 0.48). Analysis using the Breast Cancer Gene-Expression Miner v4.1(bc-GenExMiner v4.1) database showed that high P4HA2 mRNA is associated with higher metastatic relapse and/or death (p < 0.0001). Similar associations with aggressive clinico-pathologic features were observed when evaluating P4HA2 mRNA level in the invasive tumours of the METABRIC series (*n* = 1980), for example with high tumour grade (*p* = 0.03), lymph node metastasis (*p* = 0.028), ER negativity (*p* = 0.0001), HER2 positivity (*p* < 0.0001) in addition to shorter breast cancer specific survival (BCSS) (HR = 1.3, 95%CI = 1.1–1.6, *p* = 0.002). Multivariate analysis showed that higher P4HA2 mRNA level was independently associated with shorter BCSS (Supplementary Tables 3, 4 and Supplementary Figure 2).

### Outcome analysis in pure DCIS cohort

**Fig. 2:**
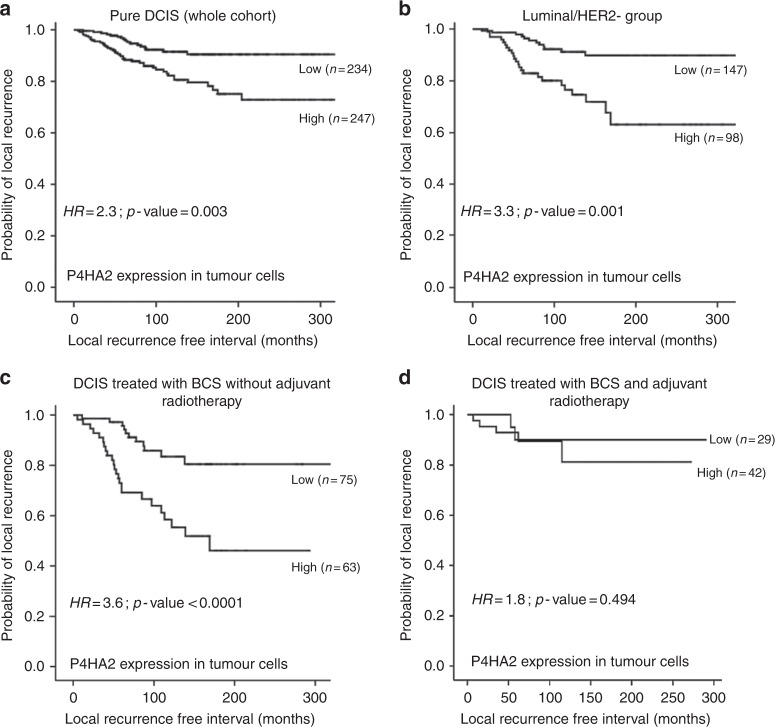
Kaplan Meier curves show that high expression of P4HA2 within the tumour epithelial cells is associated with shorter ipsilateral local recurrence free survival (LRFS) in the whole series (**a**), and in luminal/HER2- subgroup (**b**). High expression also showed an association with shorter LRFI in patients treated with breast conserving surgery (BCS) without adjuvant radiotherapy (**c**) but not in patients treated with breast conserving surgery followed by adjuvant radiotherapy (**d**)

**Fig. 3:**
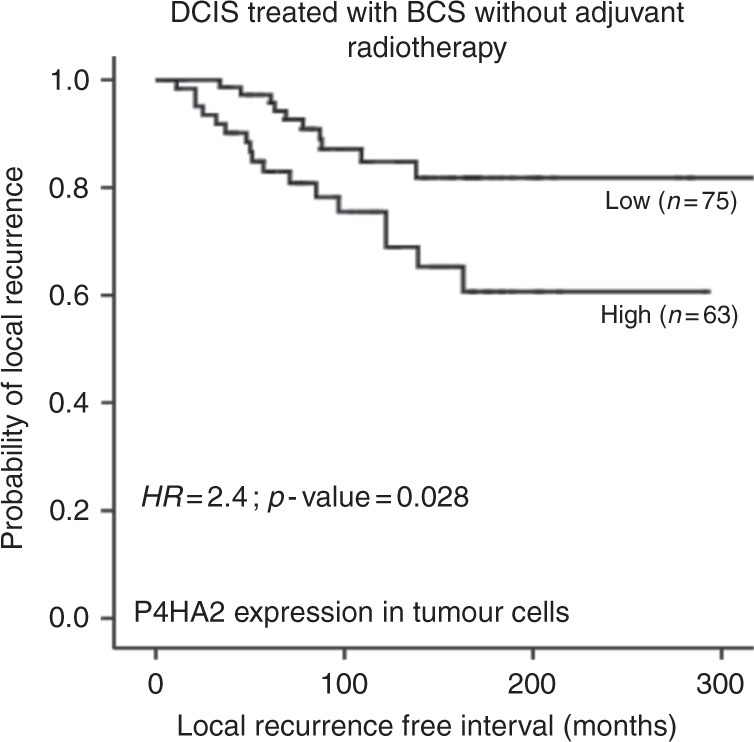
Kaplan–Meier curve shows association between high P4HA2 expression within the DCIS malignant epithelial cells and ipsilateral local recurrences as invasive carcinoma in patients treated with breast conserving surgery alone (BCS)

High P4HA2 expression within tumour epithelial cells was associated with shorter LRFI in the whole cohort of pure DCIS (HR = 2.3, 95%CI = 1.3–4.1; *p* = 0.003, Fig. 2) and in the luminal ER-positive/HER2-negative subgroup (HR = 3.3, 95%CI = 1.1–5.2; *p* = 0.001). Association with shorter LRFI was observed in patients treated with BCS without adjuvant radiotherapy (RT) (HR = 3.6, 95%CI = 1.9–7.1; *p* < 0.0001), however; the significant association with poor outcome was not maintained in patients treated with either mastectomy or BCS followed by adjuvant RT (HR = 0.9, 95%CI = 0.2–4.8; *p* = 0.9 and HR = 1.8, 95%CI = 0.3–9.1; *p* = 0.5, respectively). Interestingly, there was an association between high P4HA2 expression and ipsilateral local recurrence as invasive disease in patients treated with BCS without post-operative adjuvant RT (HR = 2.4, 95%CI = 1.1–5.2; *p* = 0.03) but this associated lost its significance in patients who were offered adjuvant RT (Figs. 2 and 3).

**Fig. 4:**
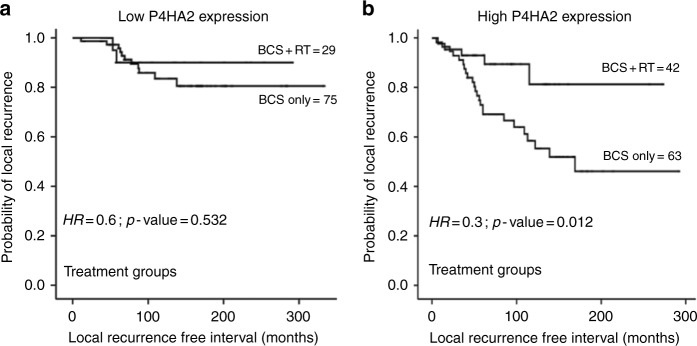
Kaplan Meier curves show association between post-operative adjuvant radiotherapy (RT) and recurrence within low and high P4HA2 expression groups; (**a**) in low P4HA2 expression group, (**b**) longer LRFI in patients treated with RT following breast conserving surgery (BCS) in high P4HA2 expression group

To further evaluate the impact of P4HA2 on the outcome in the context of adjuvant RT response, the cohort of pure DCIS treated with BCS was stratified based on P4HA2 expression. In the high P4HA2 expression group, there was a statistically significant association between adjuvant RT and longer LRFI (HR = 0.3, 95%CI = 0.1–0.8; *p* = 0.01). In the low P4HA2 expression cohort, this association was lost (HR = 0.6, 95%CI = 0.1–2.8; *p* = 0.5) (Fig. 4). The interaction between the combined RT and P4HA2 expression and outcome in the cox regression model showed similar results, whereas RT*P4HA2 expression showed a significant association with outcome (*p* = 0.01, HR = 3.4, 95%CI = 1.3–8.7).

Stromal expression of P4HA2 did not show any significant association with tumour recurrence. Forest plots illustrating the association between different clinicopathological parameters and ipsilateral local tumour recurrence are shown in Supplementary Figure 3a (all recurrences either DCIS or IBC) and Supplementary Figure 3b (invasive recurrences only).

Multivariate survival analysis showed that tumour expression of P4HA2 is a poor prognostic factor for tumour recurrence in patients treated with BCS independent of known other determinants of high risk DCIS including patient age, nuclear grade, tumour size, presence of comedo necrosis and RT in the model (HR = 2.3, 95% CI = 1.3–4.4; *p* = 0.007) (Table 2).

**Table 2:** Multivariate survival analysis (Cox regression model) of variables predicting outcome in terms of ipsilateral local recurrence in patients treated by breast conserving surgery in pure DCIS cohort

Parameters	Hazard ratio (HR)	95.0% confidence interval (CI)		Significance *p* value
		Lower	Upper	
High P4HA2 expression	3.1	1.5	6.3	**0.002**
Patient Age	0.6	0.2	1.5	0.286
DCIS presentation	1.5	0.7	2.9	0.278
DCIS size	1.3	0.7	2.5	0.437
DCIS nuclear Grade	1.3	0.7	2.3	0.373
Comedo necrosis	1.1	0.5	2.1	0.895
Molecular classes	0.8	0.5	1.1	0.170
Radiotherapy	0.3	0.1	0.8	**0.015**
Margin status	0.8	0.5	1.4	0.510

Significant *p* values are in bold

*P4HA2* prolyl-4-hydroxlase alpha subunit 2, *DCIS* ductal carcinoma in situ

Furthermore, when patients treated with BCS were stratified based on clinicopathological variables into high/intermediate (higher) risk and low risk groups, using the Van Nuys Prognostic Index,^33^ the ipsilateral local recurrence rate was comparable (12 and 10% for the higher and low risk groups; respectively). When P4HA2 expression was incorporated, the recurrence rate for the higher risk/high P4HA2 group (35% of the final cohort; i.e. 168/481 patients) was 16% but only 8% for the higher risk/low P4HA2 group. Interestingly, the low risk/high P4HA2 group (15% of the final cohort) showed a 13% ipsilateral local recurrence rate, while the low risk/low P4HA2 group had a recurrence rate of 6%. Further categorisation, in context of post-operative RT, showed that the recurrence rate in higher risk/high P4HA2 group with no RT is 25% compared to 6% for higher risk/high P4HA2 patients who received RT. Similarly, there was 19% ipsilateral local recurrence rate in low risk/high P4HA2 patients who did not receive RT compared to 5% in patients who offered adjuvant RT. The recurrence rate in low risk/low P4HA2 group with or without post-operative RT was comparable (3 and 4%, respectively). These results indicate that P4HA2 is a promising marker for better DCIS risk stratification and hence better personalised management.

## Discussion

Despite breakthroughs in various molecular techniques, predictors for DCIS aggressiveness remain elusive. Lack of robust prognostic markers is due to lack of adequately powered and methodologically sound studies (case-control, unbiased, comparison between DCIS that recur as invasive disease vs. those do not). DCIS progression is a complex process with interaction between tumour cells and microenvironment. An explanation of disease progression based exclusively on intrinsic tumour cell factors is insufficient, as there is group of low grade DCIS with indolent appearance and low proliferation index that yet carries progression potential to IBC.^34^ In contrast, a considerable proportion of high-grade proliferating lesions remain as DCIS without progression. Laser-capture microdissection and microarray analysis show that whereas thousands of genes are differentially expressed in the epithelium during the transition from normal to DCIS, the majority of genes consistently showing differential expression from DCIS to IBC are associated with the stromal microenvironment, highlighting its profound importance in the development of IBC.^12,35–38^ It was reported that changes in the breast tumour microenvironment are often observed as early as the DCIS stage or even earlier, where hyperactive mitogenic signalling in epithelial cells results in secretion of many chemokines which modulate the surrounding tissues in a paracrine-like action.^39,40^ In turn, the stromal and epithelial cells participate in reciprocal and paracrine-acting signalling loops, which then remodel and condition the ECM and promote tumour cell proliferation, maintenance and invasion.^12,14,41,42^

P4HA2 is one of the key regulator enzymes for collagen biosynthesis, stabilisation, ECM remodelling and stiffness^18^ and has been reported to associate with poor outcome in many malignant tumours.^19,20,23^ Using the METABRIC cohort for robust molecular data in a large number of IBC showed associations between aggressive behaviour of IBC and higher levels of P4HA2 mRNA. This observation supports our hypothesis that P4HA2 is a promising candidate marker for evaluation as studies to decipher its role in DCIS behaviour and its association with disease progression have been lacking.

In our study, we evaluated the pattern of P4HA2 expression either within tumour epithelial cells or surrounding fibroblasts in a large annotated cohort of DCIS with long term follow up data. This is the first report of IHC staining for P4HA2 in any human breast tumour cohort to our knowledge. High expression of P4HA2 was associated with other determinants of high risk DCIS and poor outcome. These associations give insight for the potential role of P4HA2 in DCIS behaviour through its action in enhancing stromal stiffness and collagen deposition/alignment, which in turn might help in tumour aggressiveness.^43^ Supporting this idea, our results showed that P4HA2 expression is higher in DCIS co-existing with invasive carcinoma than pure DCIS, and much higher in the stromal fibroblasts surrounding the invasive component. These findings are further supported by gene expression profiles that showed P4HA2 mRNA level is higher in IBC than DCIS.^22,23^ Stromal fibroblasts as well as tumour cells can produce P4HA2 and affect the surrounding ECM.^21^ Our data showing strong tumour epithelial cell expression is entirely consistent with this report.

Importantly, our data showed association of higher P4HA2 expression with invasive recurrence. In concordance with our findings, thickening and linearisation of collagen fibres are often found in areas of active tissue invasion, suggesting their active role in facilitating cancer progression.^17^ Indeed, studies using live imaging have shown that cancer cells migrate rapidly in areas enriched in collagen.^44^ Gilkes *et al*., reported that cancer cell invasion usually occurs with oriented collagen fibres at the tumour-stromal interface, and aligned collagen fibres can facilitate cell migration and invasion.^21^ Moreover, in xenograft models a significant amount of aligned collagen fibres is detected in the tumours’ invasion margins in a control group compared to the P4HA2-silenced group.^23^ Taken together, DCIS with high P4HA2 expression may have a microenvironment supportive of tumour growth and therefore need to be managed properly to avoid progression or recurrence. Our study shows that expression of P4HA2 in the tumour, but not stromal expression, is associated with recurrence a finding that might reflect the potential epithelial cell-intrinsic role of early stage tumours in ECM remodelling that facilitates tumour progression and the dual role of tumour and stromal cells in progression and aggressiveness of advanced tumours. The latter interaction is supported by the dramatic increase of P4HA2 expression in stromal cells surrounding the invasive component compared to those surrounding the DCIS component in mixed cases or those surrounding pure DCIS. However, further functional studies are highly recommended to understand the underlying mechanisms of P4HA2 expression in carcinogenesis and tumour progression either from the tumour cells or the surrounding stroma.

Interestingly, our results show that high P4HA2 expression is not associated with recurrence in patients treated with postoperative radiotherapy. Moreover, postoperative RT is associated with longer LRFI in patients with high P4HA2 than those with low P4HA2 expression. Taken together, P4HA2 is not only a marker to identify high-risk patients who need proper treatment with surgery followed by radiation, but also suggest that adjuvant RT provides more benefit in DCIS expressing high levels of P4HA2, which needs further investigation to understand the underlying mechanisms.

Nonetheless, multivariate analysis shows that only RT and P4HA2 expression were significant prognostic factors, suggesting P4HA2 may be a more powerful marker of high risk than conventional features. Moreover, in our cohort, using the conventional clinicopathological features and the previously described Van Nuys Prognostic Index^33^ could not provide a proper DCIS risk stratification in term of disease recurrence, which supports the contention that more robust criteria for DCIS risk determination is required. Incorporation of P4HA2 with the other clinicopathological factors provided a better identification of different risk groups. These findings indicate that P4HA2 is a promising marker for better definition of high risk DCIS as well as identification of a group of patients with lower risk where radiotherapy could be omitted.

Although the exact mechanism of how P4HA2 overexpression and subsequent ECM remodelling helps in tumour progression is unclear, there are many hints from existing data. Deregulation of ECM dynamics can facilitate cellular dedifferentiation and cancer stem cell expansion.^45,46^ Additionally, it disrupts tissue polarity and promotes cellular motility and tissue invasion.^47^ As a result, epithelial cells are directly affected by deregulated ECM dynamics, leading to cellular transformation and metastasis.^45,47–49^ Collagen fibre alignment plays a critical role in directing the migration of tumour cells in vivo.^50^ Moreover, increased stromal stiffness in breast cancer facilitates integrin clustering to promote focal adhesions between tumour cells and surrounding stroma, which drive invasion.^51^ Also, it has been shown in xenograft studies that P4HA2 regulates tumour growth. Silencing of P4HA2 expression or treatment with a P4HA inhibitor significantly inhibited cell proliferation and suppressed aggressive phenotypes of breast cancer cells in 3D culture, accompanied by reduced deposition of collagen I and IV.^23^

A key driver of P4HA2 and other ECM remodelling proteins is hypoxia and related factors, mainly HIF-1a,^27^ supporting our finding that high P4HA2 expression is associated with higher levels of HIF-1a. P4HA2 has been reported to co-localise with HIF-1α in peri-necrotic, i.e. hypoxic, areas within tumours.^27,52^ The hypoxia pathway has a complex role in tumour progression through enhancing angiogenesis, tumour proliferation, secretion of growth factors and other proteolytic enzymes.^52^ Therefore, P4HA2 overexpression might be a consequence of hypoxia related changes. In addition, hypoxia is associated with overexpression of lysyl oxidases, proteins known to promote cell invasion by increasing tissue tension and ECM rigidity.^23^ These results indicate that the ECM microenvironment remodelled by cancer cells is critical for cancer progression.

## Conclusion

ECM remodelling plays a crucial role in tumour progression. P4HA2 might have a potential role in DCIS aggressiveness through its regulatory role in collagen biosynthesis and enhancing ECM stiffness. Hypoxia and related factors could be the key drivers of such pathway. More functional studies to decipher the role of P4HA2 and its mechanism of action in DCIS behaviour are warranted. P4HA2 may also be a valuable prognostic indicator, particularly in the ER + /HER2− luminal tumours for which a biomarker that could prevent over treatment, i.e. avoid radiotherapy, is urgently required.

### Limitations of the study

This study has been carried out on TMA sections, which might underestimate the role of tumour heterogeneity. However, all cases in our cohort were histologically reviewed before TMA construction and used multiple cores for cases with heterogeneous grades or morphological patterns. Additionally, staining of some full-face sections was performed to assess the pattern of protein distribution and no obvious staining heterogeneity was observed. Lack of data for some potential confounders such as family history of breast cancer or obesity is also a limitation. Our cohort did not include any patients treated with endocrine therapy. Finally, the current study addressed the prognostic significance of P4HA2 in DCIS, but more functional mechanistic studies are highly recommended to decipher its actual role in carcinogenesis and DCIS progression as well as its interaction with radiotherapy. Evaluation the biological and prognostic roles of P4HA2 in larger validation cohorts of different DCIS subgroups, for instance, high risk, low risk DCIS, patients treated with BCS and adjuvant radiotherapy with long term follow up period would provide better understanding for such interaction.

## Electronic supplementary material

Supplementary Tables

Supplementary Figures

## Availability of data and material

The datasets generated during and/or analysed during the current study are available from the corresponding authors on reasonable request.

## References

[1] Ringberg A (2007). Histopathological risk factors for ipsilateral breast events after breast conserving treatment for ductal carcinoma in situ of the breast--results from the Swedish randomised trial. Eur. J. Cancer.

[2] Liu Y (2010). A longitudinal study of factors associated with perceived risk of recurrence in women with ductal carcinoma in situ and early-stage invasive breast cancer. Breast Cancer Res. Treat..

[3] Leonard GD, Swain SM (2004). Ductal carcinoma in situ, complexities and challenges. J. Natl. Cancer Inst..

[4] Groen Emma J., Elshof Lotte E., Visser Lindy L., Rutgers Emiel J. Th., Winter-Warnars Hillegonda A.O., Lips Esther H., Wesseling Jelle (2017). Finding the balance between over- and under-treatment of ductal carcinoma in situ (DCIS). The Breast.

[5] Knopfelmacher A, Fox J, Lo Y, Shapiro N, Fineberg S (2015). Correlation of histopathologic features of ductal carcinoma in situ of the breast with the oncotype DX DCIS score. Mod. Pathol..

[6] Carraro, D. M., Elias, E. V. & Andrade, V. P. Ductal carcinoma in situ of the breast: morphological and molecular features implicated in progression. *Biosci. Rep*. **34**, e00090 (2013). 10.1042/BSR20130077 PMC3894794 27919043

[7] Selvi Radhakrishna (2015). Breast Diseases.

[8] Zhou W (2013). Molecular subtypes in ductal carcinoma in situ of the breast and their relation to prognosis: a population-based cohort study. BMC Cancer (Res. Support, Non-U. S. Gov’t).

[9] Solin LJ (2013). A multigene expression assay to predict local recurrence risk for ductal carcinoma in situ of the breast. J. Natl. Cancer Inst..

[10] Allen MD (2014). Altered microenvironment promotes progression of preinvasive breast cancer: myoepithelial expression of alphavbeta6 integrin in DCIS identifies high-risk patients and predicts recurrence. Clin. Cancer Res..

[11] Boudreau A, van’t Veer LJ, Bissell MJ (2012). An “elite hacker”: breast tumors exploit the normal microenvironment program to instruct their progression and biological diversity. Cell Adhes. Migr..

[12] Ma XJ, Dahiya S, Richardson E, Erlander M, Sgroi DC (2009). Gene expression profiling of the tumor microenvironment during breast cancer progression. Breast Cancer Res..

[13] Thompson E (2016). The immune microenvironment of breast ductal carcinoma in situ. Mod. Pathol..

[14] Bissell Mina J, Hines William C (2011). Why don't we get more cancer? A proposed role of the microenvironment in restraining cancer progression. Nature Medicine.

[15] Kim HJ, Park CI, Park BW, Lee HD, Jung WH (2006). Expression of MT-1 MMP, MMP2, MMP9 and TIMP2 mRNAs in ductal carcinoma in situ and invasive ductal carcinoma of the breast. Yonsei Med J..

[16] Coussens LM, Fingleton B, Matrisian LM (2002). Matrix metalloproteinase inhibitors and cancer: trials and tribulations. Science.

[17] Provenzano PP (2006). Collagen reorganization at the tumor-stromal interface facilitates local invasion. BMC Med..

[18] Myllyharju Johanna (2003). Prolyl 4-hydroxylases, the key enzymes of collagen biosynthesis. Matrix Biology.

[19] Chang KP (2011). Identification of PRDX4 and P4HA2 as metastasis-associated proteins in oral cavity squamous cell carcinoma by comparative tissue proteomics of microdissected specimens using iTRAQ technology. J. Proteome Res..

[20] Jarząb B (2005). Gene expression profile of papillary thyroid cancer: sources of variability and diagnostic implications. Cancer Res..

[21] Gilkes DM (2013). Collagen prolyl hydroxylases are essential for breast cancer metastasis. Cancer Res..

[22] Pan PW, Zhang Q, Bai F, Hou J, Bai G (2012). Profiling and comparative analysis of glycoproteins in Hs578BST and Hs578T and investigation of prolyl 4-hydroxylase alpha polypeptide II expression and influence in breast cancer cells. Biochem. Biokhimiia.

[23] Xiong G, Deng L, Zhu J, Rychahou PG, Xu R (2014). Prolyl-4-hydroxylase α subunit 2 promotes breast cancer progression and metastasis by regulating collagen deposition. BMC Cancer.

[24] Hammond ME, Hayes DF, Wolff AC, Mangu PB, Temin S (2010). American society of clinical oncology/college of american pathologists guideline recommendations for immunohistochemical testing of estrogen and progesterone receptors in breast cancer. J. Oncol. Pract..

[25] Rakha EA (2015). Updated UK Recommendations for HER2 assessment in breast cancer. J. Clin. Pathol..

[26] Gong Y (2009). Performance of chromogenic in situ hybridization on testing HER2 Status in breast carcinomas with chromosome 17 polysomy and equivocal (2 + ) herceptest results: a study of two institutions using the conventional and new ASCO/CAP scoring criteria. Am. J. Clin. Pathol..

[27] Gilkes, D. M., Bajpai, S., Chaturvedi, P., Wirtz, D. & Semenza, G. L. Hypoxia-inducible factor 1 (HIF-1) promotes extracellular matrix remodeling under hypoxic conditions by inducing P4HA1, P4HA2, and PLOD2 expression in fibroblasts. *J. Biol. Chem.* **288**, 10819 (2013). 10.1074/jbc.M112.442939 PMC3624462 23423382

[28] Mittal K (2017). Amplified centrosomes and mitotic index display poor concordance between patient tumors and cultured cancer cells. Sci. Rep..

[29] McCarty KS, KS McCarty (1984). Histochemical approaches to steroid receptor analyses. Semin. Diagn. Pathol..

[30] Hendry Shona, Pang Jia-Min B., Byrne David J., Lakhani Sunil R., Cummings Margaret C., Campbell Ian G., Mann G. Bruce, Gorringe Kylie L., Fox Stephen B. (2017). Relationship of the Breast Ductal Carcinoma In Situ Immune Microenvironment with Clinicopathological and Genetic Features. Clinical Cancer Research.

[31] Camp RL, Dolled-Filhart M, Rimm DL (2004). X-tile: a new bio-informatics tool for biomarker assessment and outcome-based cut-point optimization. Clin. Cancer Res..

[32] Curtis C (2012). The genomic and transcriptomic architecture of 2,000 breast tumours reveals novel subgroups. Nature.

[33] Silverstein MJ (2003). The University of Southern California/Van Nuys prognostic index for ductal carcinoma in situ of the breast. Am. J. Surg..

[34] Afghahi A (2015). Chromosomal copy number alterations for associations of ductal carcinoma in situ with invasive breast cancer. Breast Cancer Res..

[35] Gorringe KL, Fox SB (2017). Ductal carcinoma in situ biology, biomarkers, and diagnosis. Front. Oncol..

[36] Allinen M (2004). Molecular characterization of the tumor microenvironment in breast cancer. Cancer Cell..

[37] Hu M (2008). Regulation of in situ to invasive breast carcinoma transition. Cancer Cell..

[38] Unsworth A, Anderson R, Britt K (2014). Stromal fibroblasts and the immune microenvironment: partners in mammary gland biology and pathology?. J. Mammary Gland Biol. Neoplasia.

[39] Liu S (2011). Breast cancer stem cells are regulated by mesenchymal stem cells through cytokine networks. Cancer Res..

[40] Rattigan Y, Hsu JM, Mishra PJ, Glod J, Banerjee D (2010). Interleukin 6 mediated recruitment of mesenchymal stem cells to the hypoxic tumor milieu. Exp. Cell Res..

[41] Hanahan D, Weinberg RA (2011). Hallmarks of cancer: the next generation. Cell.

[42] Karnoub AE (2007). Mesenchymal stem cells within tumour stroma promote breast cancer metastasis. Nature.

[43] Lu P, Weaver VM, Werb Z (2012). The extracellular matrix: a dynamic niche in cancer progression. J. Cell Biol..

[44] Conklin MW (2011). Aligned collagen is a prognostic signature for survival in human breast carcinoma. Am. J. Pathol..

[45] Raymond K, Deugnier MA, Faraldo MM, Glukhova MA (2009). Adhesion within the stem cell niches. Curr. Opin. Cell Biol..

[46] Shen Q (2008). Adult SVZ stem cells lie in a vascular niche: a quantitative analysis of niche cell-cell interactions. Cell. Stem. Cell..

[47] Feigin ME, Muthuswamy SK (2009). Polarity proteins regulate mammalian cell-cell junctions and cancer pathogenesis. Curr. Opin. Cell Biol..

[48] Condeelis J, Segall JE (2003). Intravital imaging of cell movement in tumours. Nat. Rev. Cancer.

[49] Wang W (2002). Single cell behavior in metastatic primary mammary tumors correlated with gene expression patterns revealed by molecular profiling. Cancer Res..

[50] Provenzano PP, Eliceiri KW, Keely PJ (2009). Shining new light on 3D cell motility and the metastatic process. Trends Cell Biol..

[51] Keely PJ, Fong AM, Zutter MM, Santoro SA (1995). Alteration of collagen-dependent adhesion, motility, and morphogenesis by the expression of antisense alpha 2 integrin mRNA in mammary cells. J. Cell Sci..

[52] Semenza GL (2016). The hypoxic tumor microenvironment: A driving force for breast cancer progression. Biochim. Biophys. Acta.

