# Supplementary material for: Prolyl-4-hydroxylase Α subunit 2 (P4HA2) expression is a predictor of poor outcome in breast ductal carcinoma in situ (DCIS)
Source: Br J Cancer. 2018 Nov 9;119(12):1518–26. doi: 10.1038/s41416-018-0337-x (PMC6288166; doi:10.1038/s41416-018-0337-x)
Supplement: Supplementary file 1 — Supplementary Tables [file 41416_2018_337_MOESM1_ESM.docx]

**Supplementary Table 1:** Clinicopathological parameters of the pure DCIS cohort*. The whole primary cohort including 776 cases and the final cohort including 481 cases after P4HA2 scoring are shown.

| Parameters | Whole cohort  N=776 cases  N (%) | Final cohort  N=481 cases  N (%) |
| --- | --- | --- |
| **Mean patient age (years ± SD)** | 57 ± 9 (range 28-86) | 57 ± 10 (range 28-86) |
| **Mean DCIS size (mm ± SD)** | 30 ± 26 (range 1-180) | 35 ± 28 (range 1-180) |
| **Patient age**  ≤45 years  >45 years | 78 (10)  698 (90) | 58 (11)  423 (89) |
| **DCIS Presentation**  Screening  Symptomatic | 421 (54)  355 (46) | 238 (50)  243 (50) |
| **DCIS Size**  ≤20mm  >20mm  N/A | 382 (49)  386 (50)  7 (1) | 204 (42)  274 (57)  3 (1) |
| **DCIS Nuclear Grade**  Low  Moderate  High | 102 (13)  192 (25)  482 (62) | 61 (13)  125 (26)  295 (61) |
| **Comedo necrosis**  Yes  No | 497 (64)  279 (36) | 321 (67)  160 (33) |
| **Surgical Management**  Breast conserving surgery (BCS)  Mastectomy | 375 (48)  401 (52) | 209 (44)  272 (56) |
| **Final Margin Status****  Positive (Tumour on ink)  <2mm  ≥2mm  Unknown | 9 (2)  11 (3)  326 (87)  29 (8) | 6 (3)  8 (4)  179 (86)  16 (7) |
| **Radiotherapy (RT)****  No  Yes | 265 (71)  110 (29) | 138 (66)  71 (34) |
| **Estrogen receptor (ER) status**  Negative  Positive  N/A | 148 (19)  439 (57)  189 (24) | 117 (24)  322 (67)  42 (9) |
| **Progesterone receptor (PR) status**  Negative  Positive  N/A | 248 (32)  336 (43)  192 (25) | 186 (39)  252 (52)  43 (9) |
| **HER2 status*****  Negative  Positive  N/A | 496 (64)  133 (17)  147 (19) | 320 (67)  108 (22)  53 (11) |
| **Molecular classes**  Luminal/HER2–  Luminal/HER2+  ER-/HER2+  Triple negative  N/A | 335 (43)  60 (8)  51 (7)  80 (10)  250 (32) | 245 (51)  52 (11)  47 (10)  59 (12)  78 (16) |
| **HIF1-α expression**  High  Low  N/A | 105 (14)  364 (47)  307 (39) | 92 (19)  273 (57)  116 (24) |
| **Recurrence**  DCIS recurrence  Invasive recurrence | 30 (36)  53 (64) | 22 (39)  34 (61) |

P4HA2; prolyl-4-hydroxlase alpha subunit 2, DCIS; ductal carcinoma in situ, HER2; Human epidermal growth factor receptor 2, BCS; breast conserving surgery, HIF1-a; hypoxia inducible factor 1 alpha.

*Mixed DCIS-IBC cohort included 239 patients with mean age 53±9 years, 155 cases (64%) had extensive DCIS, 167 cases (69%) were of high nuclear grade and comedo type necrosis was seen in 175 cases (73%).

**For patients treated with breast conserving surgery

***HER2 final status is achieved using combination of IHC and chromogenic in situ hybridisation (CISH).

**Supplementary Table 2**: Correlation between P4HA2 expression in DCIS malignant epithelial cells and stromal fibroblasts with different clinicopathological parameters in the pure DCIS cohort using continuous data.

| Parameters | Number of cases | P4HA2 expression in tumour epithelial cells (H-score) | | P4HA2 expression in stromal fibroblasts (%) | |
| --- | --- | --- | --- | --- | --- |
|  |  | Mean Rank | *p*-value | Mean Rank | *p*-value |
| **Patient Age**  ≤45 years  >45 years | 58  423 | 286.0  234.8 | **0.008** | 288.3  234.5 | **0.005** |
| **DCIS Presentation**  Screening  Symptomatic | 238  243 | 227.8  253.9 | **0.038** | 222.1  259.5 | **0.003** |
| **DCIS Size**  ≤20mm  >20mm | 204  274 | 227.5  248.5 | 0.098 | 228.7  247.5 | 0.136 |
| **DCIS Nuclear Grade**  Low  Moderate  High | 61  125  295 | 145.1  217.9  270.6 | **<0.0001** | 201.5  219.5  258.3 | **0.002** |
| **Comedo necrosis**  Yes  No | 321  160 | 263.1  196.7 | **<0.0001** | 258.3  206.2 | **<0.0001** |
| **Estrogen receptor (ER)**  Negative  Positive | 117  322 | 290.2  194.5 | **<0.0001** | 263.3  204.3 | **<0.0001** |
| **Progesterone Receptor (PR)**  Negative  Positive | 186  252 | 258.2  190.9 | **<0.0001** | 248.8  197.8 | **<0.0001** |
| **HER2 status***  Negative  Positive | 320  108 | 200.9  254.6 | **<0.0001** | 205.7  240.5 | **0.011** |
| **Radiotherapy (RT) ****  Yes  NO | 71  138 | 123.3  95.6 | **0.002** | 125.5  94.4 | **0.0003** |
| **Treatment Group**  Mastectomy  BCS | 272  209 | 249.3  230.2 | 0.134 | 242.9  238.2 | 0.725 |
| **Molecular classes**  Luminal/HER2–  Luminal/HER2+  ER-/HER2+  Triple negative | 245  52  47  59 | 170.8  219.2  268.9  263.2 | **<0.0001** | 183.7  209.6  243.8  238.4 | **<0.0001** |
| **HIF1-a expression**  High  Low | 92  273 | 233.4  166.0 | **<0.0001** | 223.3  169.4 | **<0.0001** |
| **DCIS Type**  Pure DCIS  DCIS with IBC | 481  196 | 325.8  371.4 | **0.006** | 302.3  429.0 | **<0.0001** |

**significant *p* values are in bold**

P4HA2; prolyl-4-hydroxlase alpha subunit 2, DCIS; ductal carcinoma *in situ*, HER2; Human epidermal growth factor receptor 2, BCS; breast conserving surgery, IBC; invasive breast cancer, HIF1-a; hypoxia inducible factor 1 alpha.

*HER2 final status is achieved using combination of IHC and chromogenic *in situ* hybridisation (*CISH*).

**For patients treated with breast conserving surgery.

**Supplementary Table 3:** Correlation between *P4HA2* mRNA level and the clinicopathologic parameters in the METABRIC series of invasive breast cancers (n=1980).

| Parameter | Number of cases | Mean *P4HA2* mRNA level | *p*-value |
| --- | --- | --- | --- |
| **Patient Age**  <50 years  ≥50 years | 383  1556 | 8.3  8.3 | 0.336 |
| **Tumour Size**  ≤20 mm  >20 mm | 622  1331 | 8.3  8.3 | 0.535 |
| **Histologic Grade**  1  2  3 | 170  770  952 | 8.2  8.2  8.3 | **0.034** |
| **Lymph node metastasis**  Negative  Positive | 1035  938 | 8.2  8.3 | **0.028** |
| **Oestrogen Receptor (ER) Status**  Positive  Negative | 1506  474 | 8.2  8.4 | **0.0001** |
| **HER2 Status**  Negative  Positive | 1733  247 | 8.2  8.5 | **<0.0001** |
| **PAM50 molecular classes**  Luminal A  Luminal B  Basal-like  HER2 enriched  Normal like | 718  488  329  240  199 | 8.2  8.3  8.3  8.4  8.2 | **0.005** |

**significant *p* values are in bold**

METABRIC: Molecular Taxonomy of Breast Cancer International Consortium

**Supplementary Table 4:** Multivariate survival analysis (Cox regression model) of variables predicting breast cancer specific survival in METABRIC data.

| Parameters | Hazard ratio (HR) | 95.0% confidence interval (CI) | | Significance  p-value |
| --- | --- | --- | --- | --- |
|  |  | Lower | Upper |  |
| High P4HA2 mRNA expression | 1.3 | 1.1 | 1.5 | **0.007** |
| Patient Age | 1.0 | 0.8 | 1.3 | 0.994 |
| Tumour Grade | 1.3 | 1.1 | 1.6 | **0.004** |
| Tumour Stage | 2.1 | 1.7 | 2.6 | **0.0001** |
| Lymph node metastasis | 1.4 | 1.1 | 1.9 | **0.010** |
| Molecular classes | 1.1 | 1.1 | 1.2 | **0.028** |

**significant *p* values are in bold**

METABRIC: Molecular Taxonomy of Breast Cancer International Consortium
