# Supplementary material for: Prolyl-4-hydroxylase Α subunit 2 (P4HA2) expression is a predictor of poor outcome in breast ductal carcinoma in situ (DCIS)
Source: Br J Cancer. 2018 Nov 9;119(12):1518–26. doi: 10.1038/s41416-018-0337-x (PMC6288166; doi:10.1038/s41416-018-0337-x)
Supplement: Supplementary file 2 — Supplementary Figures [file 41416_2018_337_MOESM2_ESM.docx]

**Supplementary Figures**

| **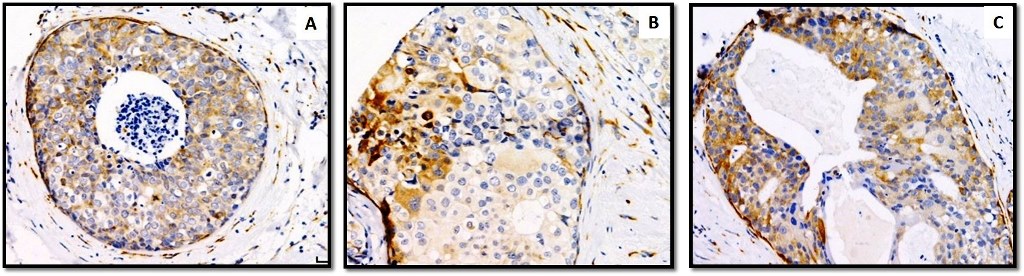** |
| --- |

**Supplementary Figure 1**: Examples of H score around 40 for P4HA2 expression in DCIS tumour epithelial cells.

| 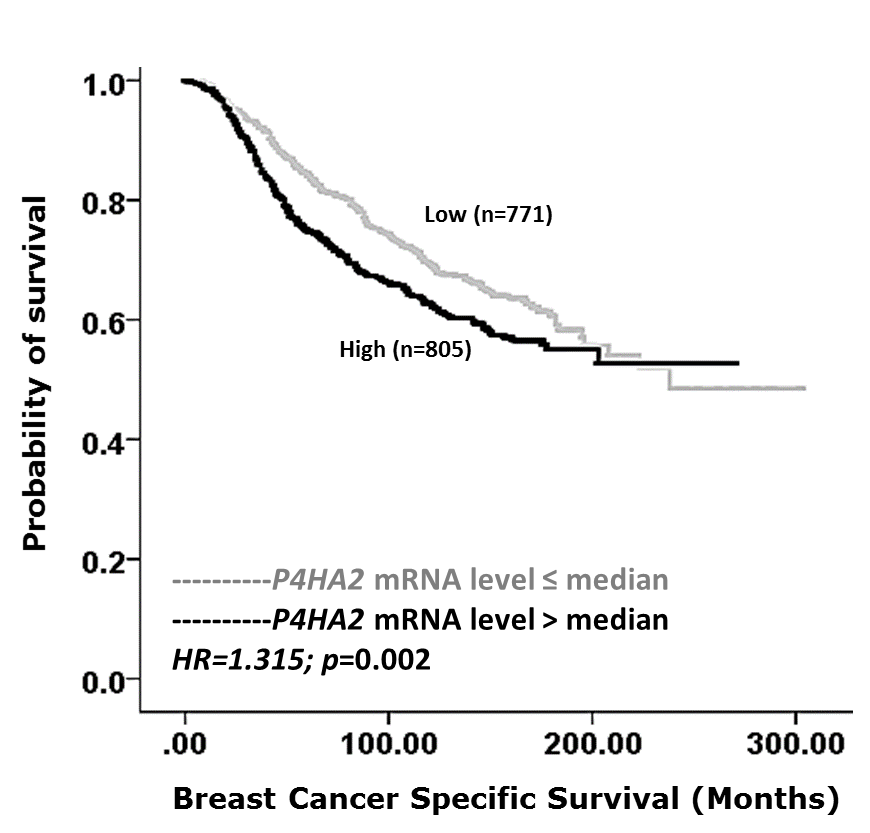 |
| --- |

**Supplementary Figure 2**: Association between *P4HA2* mRNA level and outcome in terms of breast cancer specific survival (BCSS) in the METABRIC series. The cohort was split into high and low mRNA expression based on the median (=8.28).

| 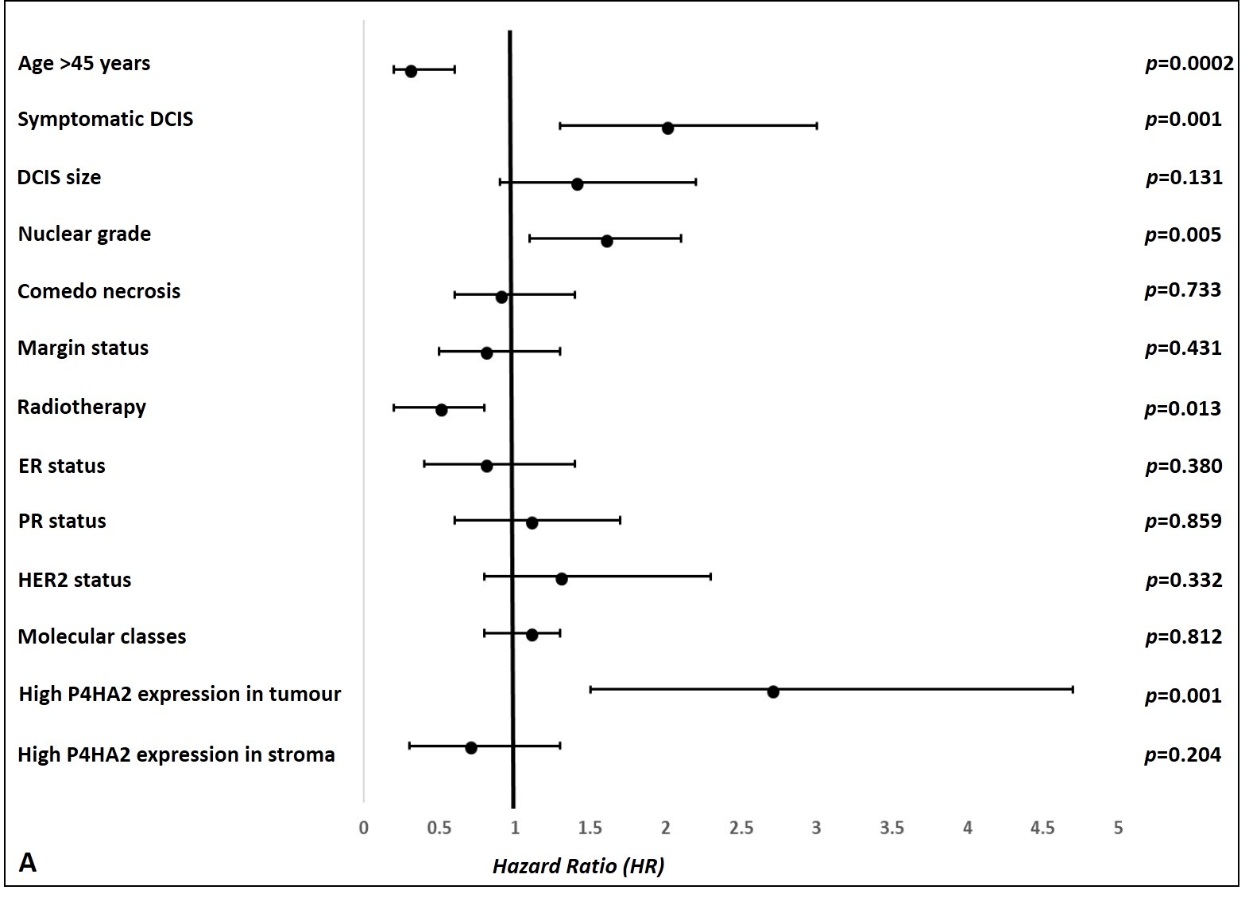 |
| --- |
| 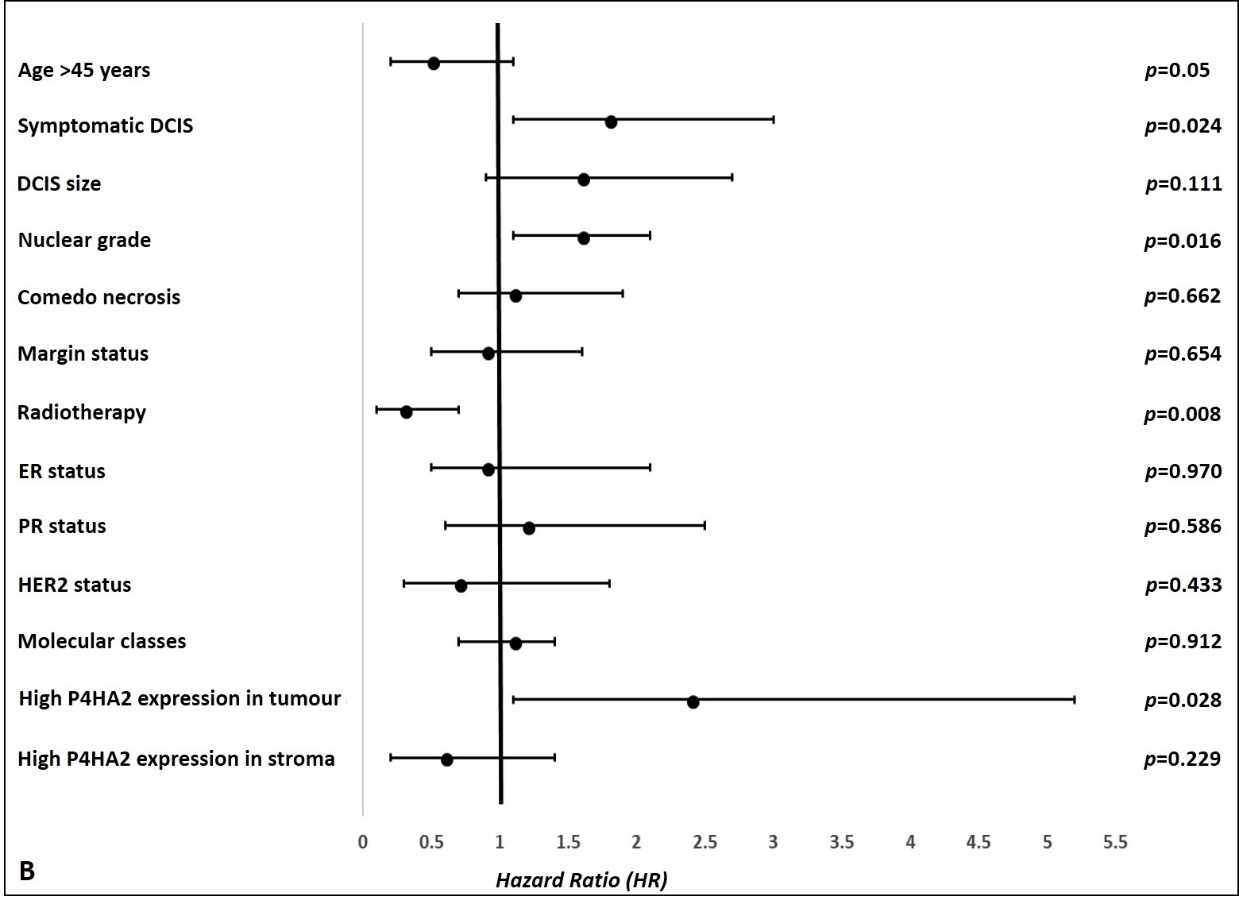 |

**Supplementary Figure 3**: Forest plots showing the univariate analysis results of association between different clinicopathological parameters and ipsilateral tumour recurrence for patients treated with **breast conserving surgery in pure DCIS cohort**; A) all recurrences whether DCIS or invasive and B) for invasive recurrences only. High expression of P4HA2 in tumour epithelial cells is associated with higher recurrence risk in both groups.
